# Giant Left Anterior Descending Coronary Artery Aneurysm in a Patient with Behçet’s Disease

**DOI:** 10.21470/1678-9741-2020-0665

**Published:** 2022

**Authors:** Mert Meric, Didem Melis Oztas, Murat Ugurlucan, Emin Tireli, Enver Dayioglu

**Affiliations:** 1 Department of Cardiovascular Surgery, Istanbul Medical Faculty, Istanbul University, Istanbul, Turkey; 2 Cardiovascular Surgery Clinic, Istanbul Training and Research Hospital, Istanbul, Turkey; 3 Department of Cardiovascular Surgery, Istanbul Medipol University Medical Faculty, Istanbul, Turkey; 4 Cardiovascular Surgery Clinic, Istanbul Medical Park Hospital, Istanbul, Turkey; 5 Cardiovascular Surgery Clinic, Gebze Merkez Prime Hospital, Istanbul, Turkey

**Keywords:** Behcet Syndrome, Coronary Aneurysm, Vasculitis, Dissection, Heart Disease Risk Factors.

## Abstract

Coronary artery involvement is quite rare in the course of Behçet’s disease. Complications secondary to coronary artery aneurysms, including rupture, dissection, and myocardial ischemia, may be fatal. In young patients without cardiovascular risk factors, systemic inflammatory vasculitis syndromes should be investigated in case of acute coronary syndrome secondary to dilated coronary arteries. In this report, we present our management strategy in a 31-year-old male patient with Bechet’s disease.

**Table:** 

Abbreviations, acronyms & symbols	
LAD	= Left anterior descending coronary artery

## INTRODUCTION

Behçet’s disease is a multisystemic inflammatory disorder which mainly presents with oral ulcers, genital ulcers, and/or ocular lesions in the affected individuals, but may also involve the nervous system, musculoskeletal system, and pulmonary and cardiovascular systems. Vascular involvement during the course of Behçet’s disease has been reported at a range of 7-38%^[1]^.

Systemic inflammatory vasculitis syndromes and Behçet’s disease should be investigated in case of unexpected vascular complications at young ages as well as surprising vascular pathologies such as aneurysms at different regions of the body, in the absence of major risk factors (*e.g*., cigarette smoking, family history, hyperlipidemia, hypertension, prolonged immobilization, etc)^[2^,^3]^. In addition, presentation of previously diagnosed patients with vasculitis to the clinic with serious vascular complications is not uncommon when the disease is not in remission due to inadequate immunosuppressive use.

In this report, we present our management strategy for coronary artery aneurysm in a 31-year-old male patient with the diagnosis of Behcet’s disease following his consent.

## CASE REPORT

A 31-year-old male patient with five years history of Behcet’s disease presented to the clinic with acute onset chest pain during walk lasting for three hours. The electrocardiogram showed ST wave changes on anterior precordial leads. Echocardiography showed mildly hypokinetic anterior and lateral myocardial walls but preserved ejection fraction and good ventricular functions. Except for increased troponin-I levels, the blood tests were within normal ranges. We decided to perform coronary angiography due to the preliminary diagnosis of acute coronary syndrome.

The patient was diagnosed with Behcet’s disease five years before with typical recurrent oral aphthous ulcers, genital ulcers, vision disturbances, relapsing thrombophlebitis, positive pathergy test, and HLA-B51 positivity. He was receiving immunosuppressive therapy with colchicine (2 mg/day), methylprednisolone (1 mg/kg/day), and azathioprine (2 mg/kg/day), and also aspirin (100 mg/day); however, he confessed irregular use of the agents when his symptoms ceased. Otherwise, he was an ex-smoker, not hypertensive, diabetic, or hyperlipidemic, and without familial predisposition.

**Fig. 1:**
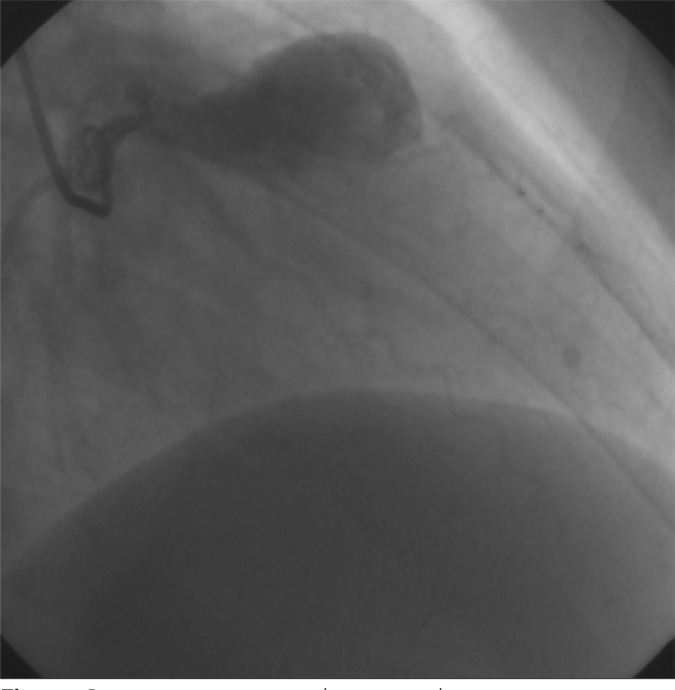
Preoperative angiographic image showing coronary artery aneurysm.

We decided to visualize the coronary arteries, and coronary angiography was planned immediately after the first dose of pulse steroid (methylprednisolone [1 g]). Coronary angiography showed giant left anterior descending coronary artery (LAD) aneurysm (Figure 1). The distal part of the LAD could hardly be visualized due to giant aneurysm sac with thrombus formations inside.

Aneurysm resection and revascularization were planned as the patient complained of chest pain on rest. Risks and benefits of the procedure and possible future complications depending on the nature of the disease were explained in detail to the patient and he was scheduled for surgery following his consent.

**Fig. 2:**
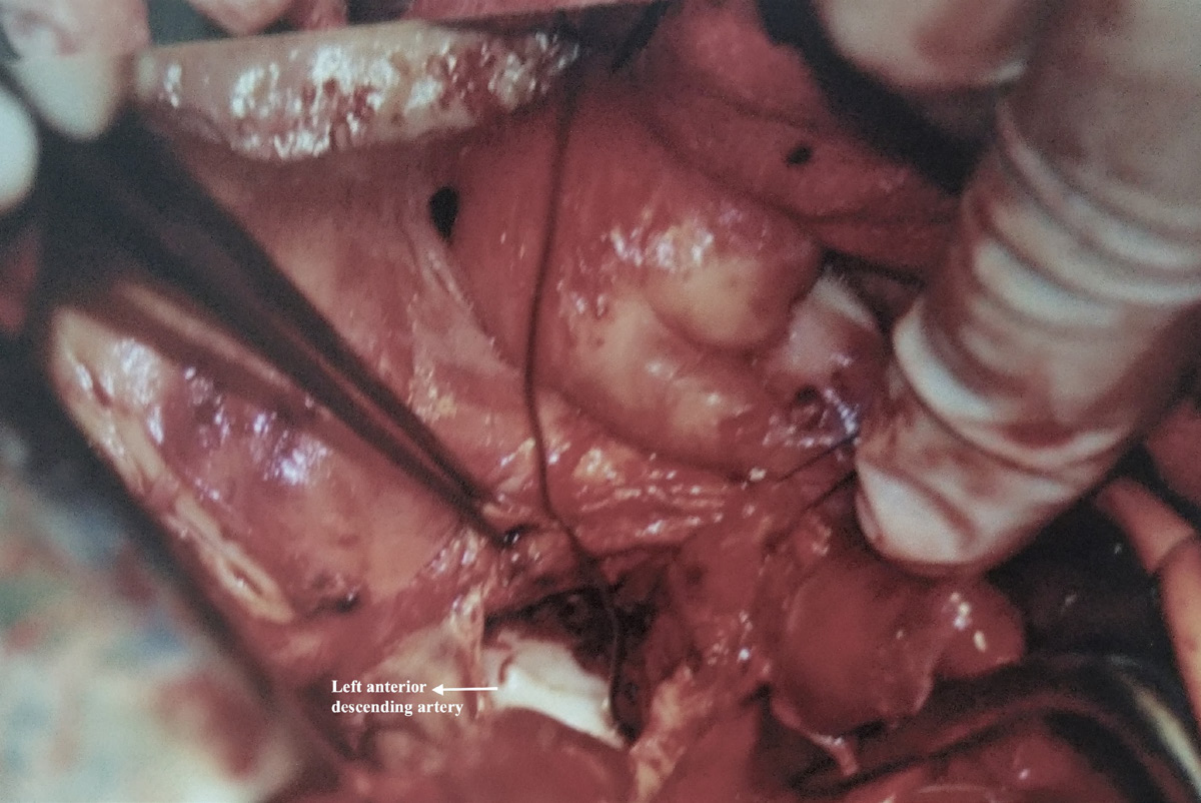
Perioperative image showing the left anterior descending artery and aneurysm sac.

After median sternotomy, the left internal thoracic artery was prepared for bypass. Aortic and two-stage atrial cannulations were performed, and cardiopulmonary bypass was initiated. Cardiac arrest was provided with antegrade cold blood cardioplegia. The aneurysm was opened. There was fresh thrombus inside the aneurysm sac (Figure 2). The proximal LAD was ligated. The left internal thoracic artery was anastomosed end-to-end to the most proximal aneurysm-free segment of the LAD. Operative and postoperative courses were uneventful with one-day stay in the intensive care unit and five-day stay in ward. His immunosuppressive therapy was adjusted accordingly at the outpatient clinic visits and he has been followed up and symptom free for more than 18 months.

## DISCUSSION

Cardiac involvement in Behçet’s disease is rare, but clearly correlated with poor prognosis^[1]^. The definite management of Behçet’s disease associated acute coronary syndrome has not been clearly identified. Medical treatment with high dose of steroids combined with immunosuppressants^[4]^, the use of thrombolytic agents^[5^,^6]^, percutaneous interventions with covered stents^[7]^, and surgical treatment are possible options for the management of the pathology. Early initiation of immunosuppressive therapy is strongly advised, and interventions such as stenting or surgery are advised after the remission of the active phase of the disease with medical suppression, due to high potency of complications^[8]^.

Being firstly described by Schiff et al.^[9]^ as myocardial infarction related to Behçet’s disease, local coronary vasculitis could result in coronary occlusion and present as acute coronary syndrome. Furthermore, inflammatory endarteritis of vasa vasorum predisposes to arterial wall weakening and aneurysm formation^[10]^. Coronary artery aneurysms, derived from Behçet’s disease, remain extremely rare, reported in < 0.5% of the patients^[11]^.

Surgical approach to coronary aneurysm consists of two techniques: aneurysmectomy with coronary artery reconstruction and aneurysm ligation with distal bypass. While planning a cardiovascular surgery in the presence of Behçet’s disease, primary repair of the affected segment should be preferred over grafting, as anastomotic pseudoaneurysms and new aneurysm formations are highly common and the most serious complications^[12]^.

However, if the use of graft material is inevitable, the graft should be carefully examined for vasculitis, and anastomosis should be carried out to the disease-free segment in order to avoid the risk of pseudoaneurysm formation and to have a better patency. Due to the large size of the aneurysm, coronary reconstruction with direct end-to-end anastomosis, avoiding grafting, was not technically possible in our case. The enhanced risk of future pseudoaneurysm formation from the anastomosis zones of graft interposition was considered, and the patient was proceeded with aneurysm ligation with distal bypass. A saphenous vein segment, other than better patency of the internal thoracic artery grafts, was not preferred because of the need for a proximal aortic anastomosis. An off-pump coronary bypass procedure would have been beneficial as it would not require ascending aortic cannulation, however, the giant size of the LAD aneurysm compromised adequate visualization of the proximal and distal disease-free segments of the vessel, hence, on-pump surgery was inevitable. Another crucial point for minimizing the risk of pseudoaneurysm formation is to avoid extra manipulations to the aorta^[13]^. Even though there is also an opposing view in the literature against the use of subclavian grafts, which emphasizes the possibility of graft occlusion secondary to vasculitis^[14]^, left internal mammary artery graft was used as recommended, instead of free vein graft, reducing the number of aortic puncture sites. Beating heart coronary artery bypass grafting procedures and sequential anastomosis are also recommended in patients with Behçet’s disease for the same reason^[13]^.

## CONCLUSION

In conclusion, a rare cause of coronary artery aneurysm originated from Behçet’s disease should not be sought in young patients without coronary risk factors in case of acute coronary syndrome, even when external disease manifestations are not present in the patient. The initiation of early immunosuppressive therapy is highly significant in preventing the disease progression, and percutaneous interventions or surgical approaches should be performed preferably after the clinical remission. However, clinical approach to the patients with critical vascular lesions and to those with ongoing ischemia despite medical treatment remains to be case oriented due to the small amount of patient population in the literature. Long-term outcomes regarding future complications and graft patency rates should be assessed in high volume multicentre studies in order to form a consensus on the clinical and surgical management of these patients.

**Table:** 

Authors' roles & responsibilities	
MM	Substantial contributions to the conception or design of the work; drafting the work or revising it critically for important intellectual content; agreement to be accountable for all aspects of the work in ensuring that questions related to the accuracy or integrity of any part of the work are appropriately investigated and resolved; final approval of the version to be published
DMO	Substantial contributions to the conception or design of the work; agreement to be accountable for all aspects of the work in ensuring that questions related to the accuracy or integrity of any part of the work are appropriately investigated and resolved; final approval of the version to be published
MU	Substantial contributions to the conception or design of the work; drafting the work or revising it critically for important intellectual content; agreement to be accountable for all aspects of the work in ensuring that questions related to the accuracy or integrity of any part of the work are appropriately investigated and resolved; final approval of the version to be published
ET	Substantial contributions to the conception or design of the work; revising the work critically for important intellectual content; agreement to be accountable for all aspects of the work in ensuring that questions related to the accuracy or integrity of any part of the work are appropriately investigated and resolved; final approval of the version to be published
ED	Substantial contributions to the conception or design of the work; revising the work critically for important intellectual content; agreement to be accountable for all aspects of the work in ensuring that questions related to the accuracy or integrity of any part of the work are appropriately investigated and resolved; final approval of the version to be published
